# Reinventing the Penumbra — the Emerging Clockwork of a Multi-modal Mechanistic Paradigm

**DOI:** 10.1007/s12975-022-01090-9

**Published:** 2022-10-11

**Authors:** Jakob Walther, Elena Marie Kirsch, Lina Hellwig, Sarah S. Schmerbeck, Paul M. Holloway, Alastair M. Buchan, Philipp Mergenthaler

**Affiliations:** 1 Charité – Universitätsmedizin Berlin, Department of Neurology with Experimental Neurology, Charitéplatz 1, 10117 Berlin, Germany; 2 Charité – Universitätsmedizin Berlin, Center for Stroke Research Berlin, Charitéplatz 1, 10117 Berlin, Germany; 3 Charité – Universitätsmedizin Berlin, NeuroCure Clinical Research Center, Charitéplatz 1, 10117 Berlin, Germany; 4 Acute Stroke Programme, Radcliffe Department of Medicine, University of Oxford, Oxford, OX3 9DU UK

**Keywords:** Stroke, Penumbra, Glucose metabolism, Mitochondria, Apoptosis, Cell death, Autophagy, Neuroinflammation

## Abstract

The concept of the ischemic penumbra was originally defined as the area around a necrotic stroke core and seen as the tissue at imminent risk of further damage. Today, the penumbra is generally considered as time-sensitive hypoperfused brain tissue with decreased oxygen and glucose availability, salvageable tissue as treated by intervention, and the potential target for neuroprotection in focal stroke. The original concept entailed electrical failure and potassium release but one short of neuronal cell death and was based on experimental stroke models, later confirmed in clinical imaging studies. However, even though the basic mechanisms have translated well, conferring brain protection, and improving neurological outcome after stroke based on the pathophysiological mechanisms in the penumbra has yet to be achieved. ﻿Recent findings shape the modern understanding of the penumbra revealing a plethora of molecular and cellular pathophysiological mechanisms. We now propose a new model of the penumbra, one which we hope will lay the foundation for future translational success. We focus on the availability of glucose, the brain’s central source of energy, and bioenergetic failure as core pathophysiological concepts. We discuss the relation of mitochondrial function in different cell types to bioenergetics and apoptotic cell death mechanisms, autophagy, and neuroinflammation, to glucose metabolism in what is a dynamic ischemic penumbra.

## Introduction

There is no sun without the shadow and it is essential to know the night.

*Albert Camus, The Myth of Sisyphus, 1942*

Ischemic stroke remains one of the leading causes of death and disabilities worldwide [1]. So far, the clinical translation of neuroprotective treatment strategies from bench to bedside has not been delivered. Nevertheless, many fundamental advances in basic understanding of cellular and molecular pathophysiological mechanisms have been made. As early as 1925, Spielmeyer observed selective, region-specific cell death of neurons in the cornu ammonis (CA)1 region of the hippocampus in a model of transient global ischemia, while other regions of the hippocampus remained intact [2]. Spielmeyer’s finding led to the concept of selective ischemic neuronal death, which was confirmed in other animal models of global ischemia in the 1970s and 1980s [3, 4].

With subsequent development of focal models of cerebral ischemia mimicking large vessel occlusion and injury to all cell types, observed in thromboembolic stroke in humans [5, 6], the concept of the ischemic penumbra around a necrotic stroke core was first discussed in 1972 and later conceptualized [7], based on the astronomical term for the shadow of a partial solar eclipse. During severe cerebral ischemia, a critical shortage of oxygen and nutrients leads to anoxic depolarization and rapid necrotic cell death in what is defined as the core. The penumbra was originally defined as a transient state of “neuronal lethargy” with electrical failure and potassium release but not terminal depolarization and ensuing neuronal cell death [8]. It is now generally considered as hypoperfused pan cellular brain tissue with decreased oxygen and glucose availability while exquisitely time sensitive, it is by definition at risk, but potentially salvageable tissue in focal stroke. The original idea of the concept celebrates its 50^th^ anniversary this year. Originally based on findings in experimental stroke models [9], later confirmed in humans in clinical imaging studies [10–12]. Although tantalizingly offering the field with a potential target for treatment [13], one that lends itself to imaging and the opportunity to rescue brain tissue not only by urgent reperfusion, but in addition by intervention in the processes committing cells to cell death pathways. However, neuroprotection of this salvageable tissue has proven elusive. Today it is clear, that the penumbra is not a single entity, but a dynamic and heterogeneous target not only through space and time, but in the heterogeneous cellular and molecular mechanisms that determine tissue fate [10, 14]. In 2001, the neuro-glial-vascular unit (NVU) was proposed as a concept that helped to overcome the dichotomy of blood vessels and cells of the brain as distinct and separate entities [15]. It facilitated the study of symbiotic inter-relationships between blood vessels and brain cells and conceptualized for the first time a multi-cellular approach to improve translational outcome in stroke. Numerous experimental studies have been conducted to achieve neuroprotection by targeting specific individual events that were discovered to occur in the penumbra after stroke, but so far none of them has led to translational success [16]. Thus, despite decades of mechanistic experimental [6] and clinical research, the only successful therapies for stroke remain the early restoration of blood flow with recombinant tissue-type plasminogen activator (rtPA) or endovascular thrombectomy [17, 18].

While the value of targeting the penumbra for therapeutic neuroprotection has not yet been realized in the clinic, its importance is evident through clinical trials of endovascular treatment in which trials selecting for patients with small infarct core and relatively large penumbral tissue have shown the highest odds for favorable functional outcome [19, 20]. As we mark 50 years since the concept, fundamental questions remain: First, what are the key underlying mechanisms in the penumbra that tip the balance for the fate of a cell to survival or cell death? Second, what must a treatment strategy for the tissue at risk be composed of in terms of targeting, timing, and duration of application, to precisely interfere with the cell death mechanisms and to confer protection of the brain in stroke?

**Fig. 1:**
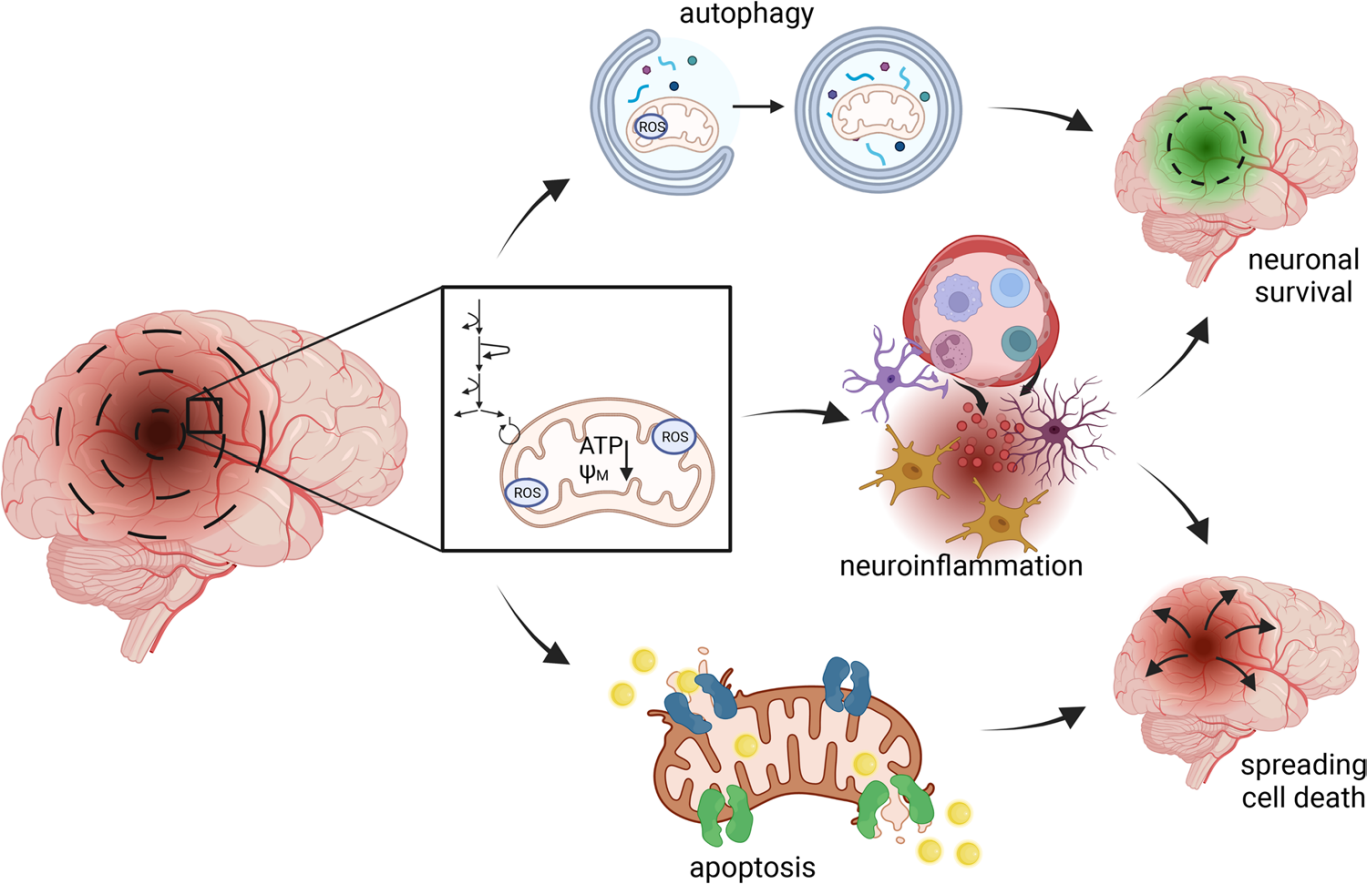
Balancing of active processes in the penumbra determine the outcome after stroke. Change in glucose and oxygen availability lead to a shift in energy homeostasis and glucose metabolism. Mitochondria set the stage for cell fate decisions and acute neurodegeneration after stroke. Opposing mechanisms like apoptosis leading to cell death, and autophagy contributing to cellular resistance are regulated by glucose metabolizing enzymes, among others. Secondary mechanisms like neuroinflammation are influenced and determined by a shift in metabolism leading to pro- or anti-inflammatory responses and subsequently contribute to stroke outcome. *ψM - mitochondiral membrane potential*

In this review, we highlight recent findings that shape our modern understanding of the penumbra and its detailed molecular and cellular pathophysiological mechanisms, and consider a multi-modal (i.e., multi-mechanistic, multi-cellular, multi time point) model as foundational for future translational success. We argue shifting the view on dynamic “states of lethargy” [8] in the penumbra with spatiotemporally interconnected mechanisms leading to cell death, rather than simply describing the penumbra as a homogeneous structure around the infarct core. Our focus is on the availability of glucose, the brain’s central source of energy, and bioenergetic failure as core pathophysiological concepts [21] which leads us to discuss the relation of mitochondrial function including bioenergetics, cell death mechanisms, autophagy, and neuroinflammation, to glucose metabolism (Fig. 1) and potential novel opportunities for therapeutic intervention.

## Mitochondrial Function in Physiological and Pathological Conditions

### Mitochondrial Bioenergetics and Metabolism

**Fig. 2:**
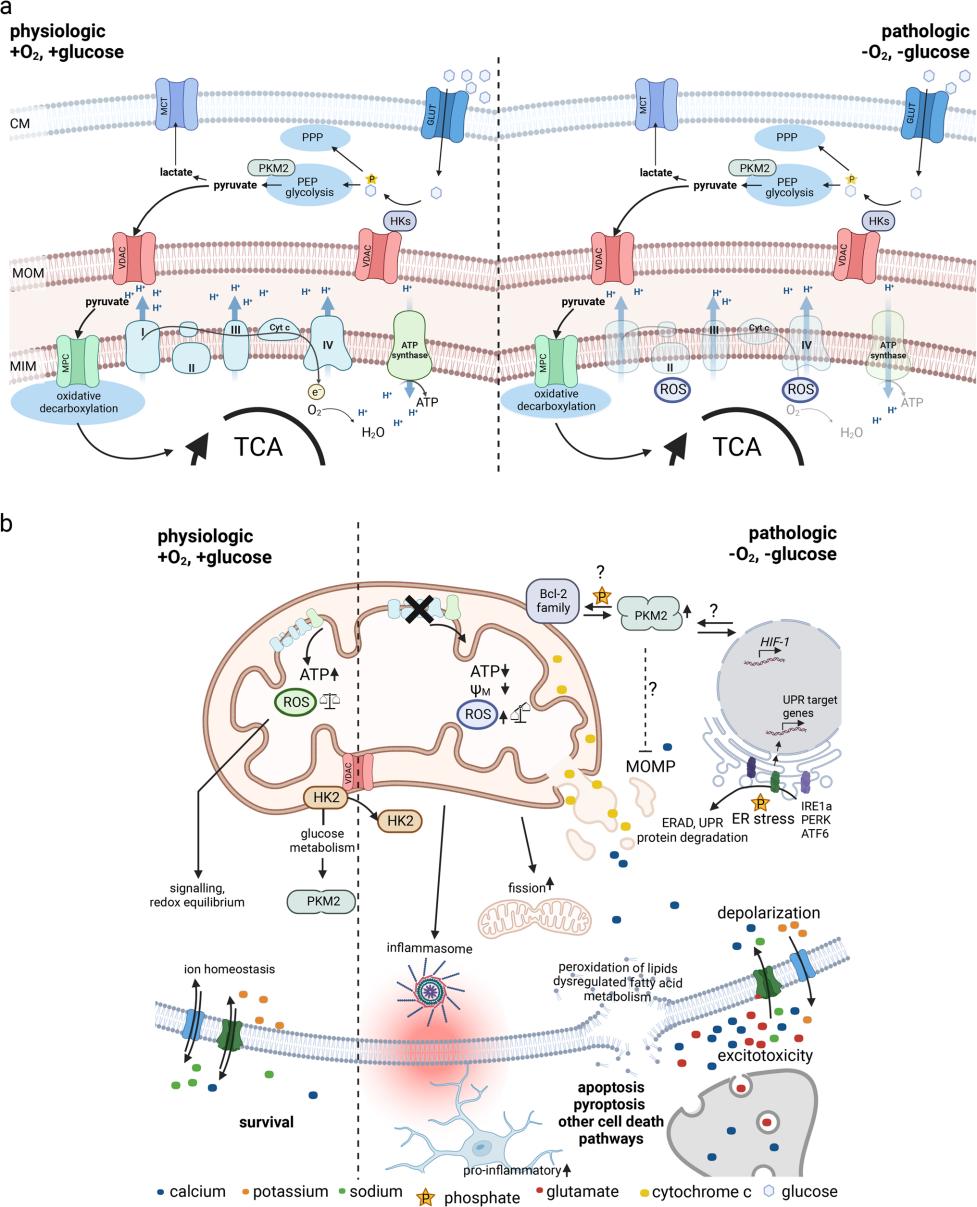
Mitochondrial function in cellular bioenergetics and metabolism. **a**, left: Under physiologic conditions, glucose is transported into the cell by glucose transporter (GLUT), phosphorylated by hexokinases (HKs) in a feedback-inhibited manner thereby trapping glucose in the cell. In the following, glucose-6-phosphate is either used for the pentose-phosphate-pathway (PPP), regenerating nicotinamide adenine dinucleotide phosphate (NADPH) and metabolic precursors or further metabolized through several reactions of glycolysis to pyruvate via phosphoenolpyruvate (PEP) catalyzed by pyruvate kinase (PK). After being transported into mitochondria via the voltage dependent anion channel (VDAC) and mitochondrial pyruvate carriers (MPCs), pyruvate is metabolized in oxidative decarboxylation providing Acetyl-CoA for the tricarboxylic acid cycle (TCA). Both provide reduction equivalents for OXPHOS in the mitochondrial inner membrane (MIM), which results in efficient adenosine triphosphate (ATP) production. Alternatively, pyruvate can be used for the generation of lactate which is exported or imported via monocarboxylate transporters (MCTs) in the cell membrane (CM) depending on cell type, energy consumption and oxygen availability. **a**, right: Upon limited availability of oxygen and glucose, as is occurring in the penumbra, bioenergetics shift from OXPHOS to glycolysis resulting in decreased ATP availability and increased lactate and ROS formation. **b** The shift from aerobic to anaerobic bioenergetics leads to a drop in ATP availability followed by insufficient ion transport, dysregulation of membrane potential and increased ROS levels. This can trigger mitochondrial outer membrane permeabilization (MOMP), cytochrome c release and thus, initiation of apoptosis. Further, imbalance of ion distribution leads to elevated glutamate release, triggering excitotoxicity. Activation of the inflammasome leads to cells death by pyroptosis and induction of inflammatory reactions. Moreover, high ROS levels lead to increased mitochondrial fission which especially in neurons leads to insufficient energy supply and cell death. High ROS levels lead to peroxidation of lipids further promoting MOMP. At the endoplasmatic reticulum (ER), phosphorylation of proteins like endoribonuclease/protein kinase Inositol-Requiring Enzyme 1α (IRE1α), Protein Kinase RNA-Like ER Kinase (PERK) and Activating Transcription Factor 6 (ATF6), and subsequent induction of transcription factors trigger mRNA and protein degradation via the unfolded protein response (UPR) or endoplasmic-reticulum-associated protein degradation at the ER (ERAD) and thus stalling protein translation and metabolism [28, 29]. Glucose-phosphorylating hexokinase II (HKII) acts as a metabolic switch. Upon glucose deprivation, HKII dissociates from voltage-dependent anion channel (VDAC) in the mitochondrial outer membrane (MOM), ultimately resulting in cell death. Alternatively, under hypoxic conditions, HKII can prevent cell death supported by phosphoprotein enriched in astrocytes (PEA15) [30] or Tp53-induced Glycolysis and Apoptosis Regulator (TIGAR). Another regulator of apoptosis in glucose metabolism is PKM2. PKM2 is upregulated upon oxygen–glucose deprivation and reperfusion and can trigger neuroprotection by preventing MOMP or by interaction and regulation of B cell lymphoma 2 (Bcl-2) family proteins like, Bcl-2, Bcl-xL or BH3-only proteins. Moreover, dimeric PKM2 has been suggested to enter the nucleus and contribute to regulating Hypoxia-Inducible Factor (HIF)-1α-dependent gene expression, among others. *ψM - mitochondrial membrane potential*

While cells in the ischemic core rapidly die by necrosis due to anoxia, cells in the penumbra experience hypoxic conditions, leading to mitochondrial dysfunction. Mitochondria are the cell’s energy-producing organelles (Fig. 2). By utilizing glucose and oxygen in oxidative phosphorylation (OXPHOS), neurons mainly rely on glucose for their energy production, and due to their high energy turnover, neurons are particularly vulnerable if energy supplies cease [21, 22]. For adenosine triphosphate (ATP) synthesis, mitochondria utilize the protein complexes forming the electron transport chain (ETC) which resides in the mitochondrial inner membrane (MIM, Fig. 2a). Catalyzing different redox reactions, the ETC establishes a proton gradient along the MIM, which is then used to produce ATP. Oxygen functions as the final electron acceptor and reactive oxygen species (ROS) are produced [23]. In the penumbra, oxygen is relatively limited which impedes the reactions of the ETC and renders a reduced state (Fig. 2a). Although moderate levels of ROS are crucial for maintenance of homeostasis and redox equilibrium as well as the regulation of neuronal activity [24], during hypoxia, the slow accumulation of ROS along with a (mitochondrial) ROS burst upon reperfusion [25] lead to oxidation of various cell compartments and membranes, loss of membrane integrity, cytoskeletal damage, and DNA damage, which in the absence of intervention lead to commitment to cell death pathways [26] and a loss of the penumbral tissue. Furthermore, calcium has been identified to play different roles in regulating glycolysis and OXPHOS after neuronal activation [27].

Mitochondrial dysfunction is one of the earliest responses to glucose / oxygen deprivation in cerebral ischemia [6, 31]. Mitochondria are directly involved in apoptotic pathways, homeostasis of reactive oxygen species, control of intracellular calcium homeostasis, and participate in important metabolic functions including nucleotide, fatty acid, amino acid, and glucose metabolism [32] (Fig. 2b).

#### Glucose Metabolism and Its Relation to Cell Death Pathways

Mitochondrial hexokinase (HK) regulates glucose metabolism by phosphorylating glucose to glucose-6-phosphate in a feedback-inhibited reaction. With this reaction, glucose is trapped in the cell and glucose-6-phosphate becomes the substrate for glycolysis, generating pyruvate which is either metabolized in the tricarboxylic acid (TCA) cycle by OXPHOS or becomes lactate in hypoxic conditions. Alternatively, glucose-6-phosphate can be processed in the pentose-phosphate pathway, generating reducing equivalents of nicotinamide adenine dinucleotide phosphate (NADPH) and building precursors for further biosynthesis, or it is used for glycogen-synthesis in astrocytes [21, 33]. Although HK1 is the major HK isoform in the brain, hypoxia-regulated HKII contributes to regulating (neuronal) cell death depending on the cellular metabolic state [21, 30]. Thus, it acts as switch to either promote pro- or anti-apoptotic pathways [30]. Glucose deprivation leads to dissociation of HKII from the mitochondria and ultimately results in cytochrome c release through mitochondrial outer membrane permeabilization (MOMP) [34]. Alternatively, HKII prevents cell death under hypoxic conditions [30, 34–36]. Reminiscent of the increased anti-apoptotic capacity of HKII through its direct interaction with phosphoprotein enriched in astrocytes (PEA15) [30], Tp53-induced Glycolysis and Apoptosis Regulator (TIGAR) increases its glycolytic capacity under hypoxic conditions [37]. Biochemically, TIGAR is a fructose-2,6-bisphosphatase which can dampen glycolysis, thereby promoting the pentose-phosphate-pathway (PPP) and leading to increased anti-oxidative NADPH levels, thereby reducing ROS-induced apoptosis [37].

Pyruvate kinase (PK) is the final and one of the rate limiting enzymes in the glycolytic cascade. PK metabolizes phosphoenolpyruvate to pyruvate in an ATP-generating reaction and is expressed in four isoforms in a tissue and context dependent pattern [38]. The PK isoform M2 (PKM2) has been shown to be involved in regulating cell death in various tissues, most prominently in cancer cells. Although the exact mechanism is still debated [38], a tetrameric form of PKM2 seems to be able to prevent MOMP by regulating mitochondrial dynamics [39] or potentially by interacting with pro-apoptotic Bcl-2-family proteins (Fig. 2b) [40]. In both in vitro and in vivo models of outer retinal apoptotic stress, a tetramerization-inducing PKM2-activating small molecule was found to inhibit apoptosis in photoreceptors [41]. Furthermore, induced tetramerization with a PKM2 inhibitor inhibited apoptosis and prevented nuclear translocation of PKM2 in human neurons derived from patients with Alzheimer’s disease [42]. PKM2 has been shown to be regulated in the brain in response to hypoxia in different settings. It has been shown to be upregulated in human cerebral organoids after oxygen–glucose deprivation/reperfusion (OGD/R) [43]. It was also found to be upregulated in neurons in a rat model of neonatal hypoxic-ischemic encephalopathy, and PKM2 knockdown decreased neuronal cell death [44]. PKM2 has been shown to be upregulated in Alzheimer’s disease in humans and in mice, and to mediate γ-secretase activity in hypoxic neurons [45]. Moreover, inhibition of PKM2 decreased cell death in neurons derived from patients with Alzheimer’s disease [42].

Finally, not only glucose metabolizing enzymes participate in cell death regulation, but also classic apoptosis-regulating proteins, such as Bad, have been shown to be involved in regulating glucose metabolism in general [46, 47] as well as neuronal excitability [48, 49] and thereby may play a role in excitation-induced neuronal cell death.

#### Lipid degradation

Fatty acids provide energy under hypoglycemic conditions and mitochondria are, together with peroxisomes, the main drivers of lipid degradation by mitochondrial β-oxidation [50, 51]. β-oxidation fuels the TCA cycle by providing acetyl-coenzyme (Co) A, and OXPHOS, by generating dihydroflavine adenine dinucleotide (FADH2) and reduced nicotinamide adenine dinucleotide (NADH). In ischemic conditions, fatty acid metabolism has been found to be dysregulated in metabolizing phosphatidylcholine as well as elevated levels of free fatty acids (lysophosphatidylcholine and diacylglycerol). Accumulation of lipid break-down products has been shown, potentially due to defective degradation pathways, contributing to cell membrane breakdown [50]. Moreover, high levels of ROS, as found during ischemia, result in peroxidation products like cyclopentenone isoprostanes, which have been found to induce mitochondrial membrane breakdown and induction of apoptosis in neurons, while inhibition of lipid peroxidation has been shown to reduce apoptosis [52, 53].

### Cell Type-Specific Effects on Mitochondrial Function in the Penumbra

Besides the described general shift from aerobic to anaerobic metabolism accompanied by depolarization and ROS formation, hypoxia also has cell specific effects on mitochondrial function in individual cell types.

#### Neurons

Neuronal activity is highly dependent on physiological mitochondrial function [21]. For this, mitochondria are efficiently distributed at the most energy demanding neuronal sites, like growth cones, presynaptic sites, or along axons and dendrites, balancing energy demand and energy supply [54]. This is achieved through tight regulation of mitochondrial motility, fusion and fission, and stationary anchoring of mitochondria to secure energy supply at the location of the highest demand [55, 56]. Neuronal activity is highly dependent on mitochondrial function regulating neurotransmitter release as well as protein synthesis and axonal transport [54]. High numbers of mitochondria in the synaptic region support release and uptake of neurotransmitters, while mitochondria in the soma are considered to be more involved in generating energy for protein synthesis and their axonal transport [54]. Since neurons are highly dependent on the availability of energy, they are heavily affected by mitochondrial dysfunction. Impaired mitochondrial motility in neurons contributes to diminished energy supply [54, 57]. The activation of the mitophagy-regulating phosphatydilinostiol-3,4,5-triphosphate 3-phosphatase (PTEN)-induced putative kinase (PINK) / Parkin pathway (see below), e.g., by increased ROS levels, leads to depletion of mitochondria from synaptic terminals by activating mitochondrial Rho GTPase 1 (Miro1) [58]. Neuronal mitochondria express prohibitin, which reduces ROS production by stabilizing complex I in the ETC to intrinsically protect neurons from an oxidative insult [59], while cell death is tightly connected to hypoxia-regulated hexokinase II and its direct interaction with PEA15 [30].

#### Neuro-Glial-Vascular Unit

The NVU is formed by brain microvascular endothelial cells (BMVECs), pericytes, astrocytes, and neurons and regulates the influx of ions, proteins, amino acids, and metabolites into the brain, maintaining homeostasis. During stroke, this sensitive system becomes dysregulated by increased ROS and calcium levels [60–62] and can also be damaged by subsequent hemorrhage [63]. BMVECs are characterized by high numbers of mitochondria and are affected by hypoxia-induced cellular stress resulting in altered blood–brain-barrier (BBB) permeability [64, 65]. Under physiological conditions, BMVECs express high amounts of tight junction proteins and are crucial for regulation of paracellular transport of water-soluble substances and ions [66, 67]. ROS dysregulate expression of tight junction proteins leading to alterations of BBB permeability and impaired barrier function [68–71]. Furthermore, excessive ROS production causes peroxidation of membrane lipids and proteins, like the Na+/K+-ATPase severely affecting BBB integrity and permeability leading to BBB dysfunction [72–74]. Ischemia modulates BBB permeability altering the passage of lipids, ions, metabolites, and cells to the brain. Furthermore, this facilitates crossing of leucocytes followed by activation of matrix metalloproteinases (MMPs), especially MMP-9, leading to degradation of both the basal lamina and tight junction proteins [75–78].

Pericytes form tight contacts with BMVECs and astrocytes regulating BBB permeability and vasoconstriction as well as providing regeneration potential to the BBB. Stroke induced oxidative stress and elevated calcium levels have been shown to induce persistent pericyte-driven constriction of collateral capillaries prolonging restriction of blood-flow and inhibiting reoxygenation [79, 80]. Moreover, pericytes actively release MMP-9, which not only leads to degradation of the basal lamina and tight junction proteins, but most importantly affects the binding of astrocyte end feet to BMVECs. Further, pericytes might support tissue repair after stroke by platelet-derived growth factor receptor ß (*PDGFR-β*) signaling [81].

Astrocytes interface between BMVECs and neurons and are crucial for neuronal energy supply by regulating cerebral blood flow together with pericytes depending on neuronal activity [82] and the distribution of metabolites [21]. In general, astrocytes have a rather anaerobic metabolism with high levels of glycolysis while in neurons, aerobic metabolism prevails. This makes astrocytes more resistant to hypoxic conditions [83] and enables them to support neuronal survival and to buffer mitochondrial induced stress, e.g., by taking up glutamate and reducing excitotoxicity [84]. Interestingly, in previous studies, it has been suggested that damaged neurons release defective mitochondria which are ultimately engulfed by astrocytes [85]. On the other hand, astrocytes have also been shown to provide functional mitochondria to neurons in stroke and other conditions [85–87]. Recently, it has been suggested that astrocytes receive mitochondria from pericytes and BMVECs by tunneling nanotubes, a process which is thought to be upregulated by hypoxia [88].

#### Microglia

Mitochondria play a pivotal role in the regulation of post-ischemic inflammation. Increasing oxidative stress by ROS and formation of free radicals during ischemic stroke leads to further accumulation of O_2_^-^ and NO_3_^-^, which leads to nitric oxide synthase (inducible, endothelial or neuronal NOS) activation, cytokine release, and an inflammatory response. Subsequently, the inflammasome is assembled at the mitochondrial outer membrane, the innate immune response is activated and pyroptosis is initiated [89]. Microglia activation is directly linked to mitochondrial function. Increased levels of mitochondrial fission in stroke direct microglia to a pro-inflammatory phenotype [90]. Dysregulation in the ETC leads to increased ROS formation and secretion of pro-inflammatory cytokines [91]. Mitochondrial ROS production leads to activation of macrophages, B and T cells [92–94].

#### Oligodendrocytes

Relatively little is known about mitochondrial function in oligodendrocytes in hypoxic conditions even though oligodendrocytes are highly vulnerable to energy deprivation and excitotoxicity depending on their maturation state [95, 96]. Mature oligodendrocytes have been shown to mainly rely on aerobic glycolysis for energy generation [97]. In oligodendrocytes, mitochondria are proposed to be ideal for calcium buffering due to their localization and their role in lipid metabolism might be crucial for maintaining the myelin sheath [98]. In general, mitochondrial length and density has been found to be lower in oligodendrocytes, compared to those in neurons suggesting a low ATP generation. Moreover, mitochondrial motility is slower in oligodendrocytes than in neurons. In contrast to neurons and astrocytes, glutamate leads to increased mitochondrial motility in oligodendrocytes [99]. In hypoxic and hypoglycemic conditions, oligodendrocytes shift to a glycolytic metabolism which favors cell survival over maintenance of the myelin sheath and thus can lead to demyelination of axons [97, 100].

In summary, mitochondria are crucial for energy generation and metabolism but can also contribute to induction of cell death pathways (see below). In the ischemic core, necrotic cell death due to acute lack of ATP and energetic crisis prevails, whereas mitochondria in the penumbra are still viable but compromised and prone to apoptosis [101, 102]. The relation between metabolic and cell death pathways and how their balance is impacted in the penumbra needs to be studied in greater detail in order to lead to novel therapeutic targets.

### The Role of Mitochondria in Cell Death Mechanisms and Reperfusion Injury

**Fig. 3:**
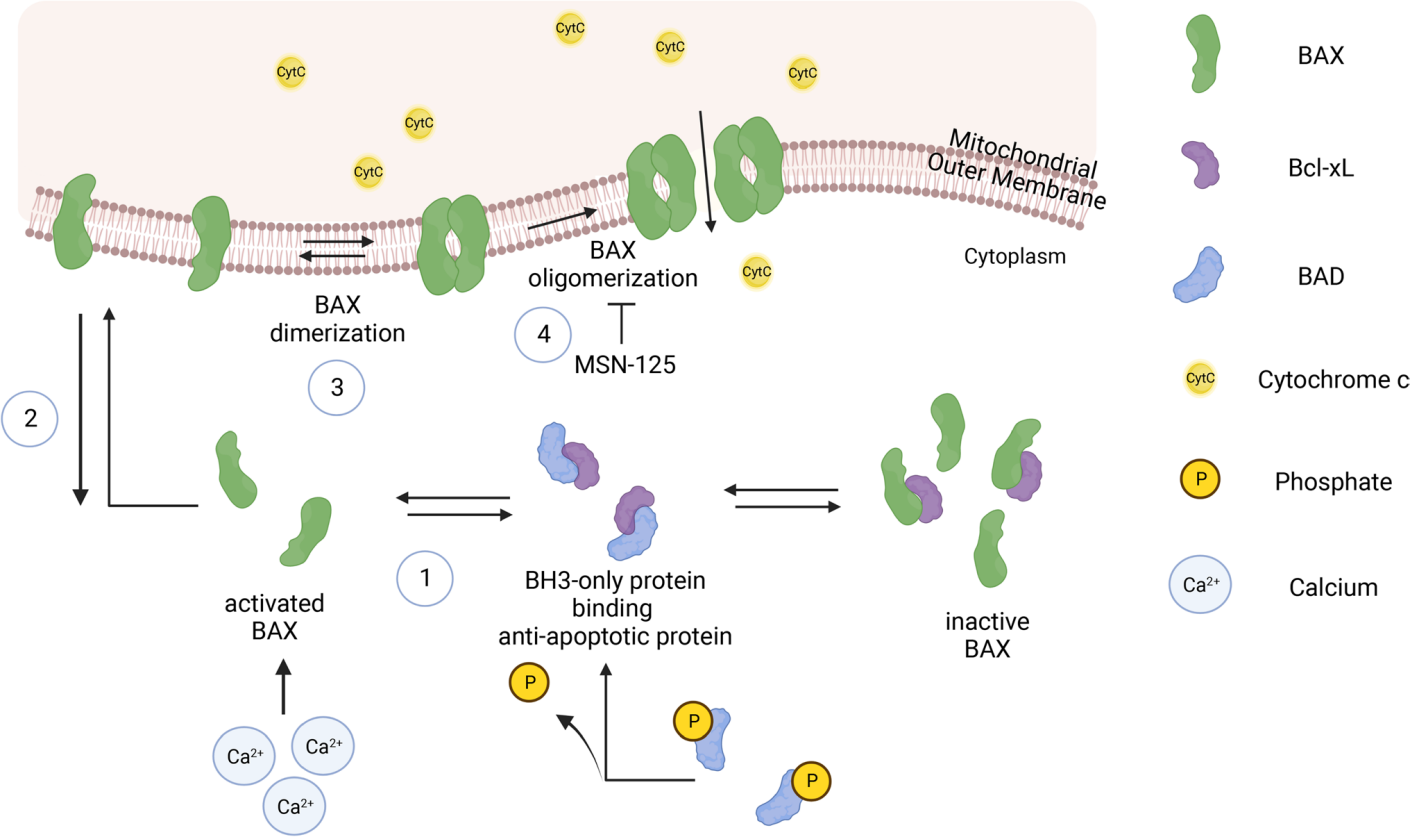
Intrinsic apoptosis pathway in the ischemic penumbra: Proteins of the Bcl2-family are the main drivers of the intrinsic apoptotic pathway. In its inactive conformation, the pro-apoptotic protein BAX (green) resides in the cytosol and is bound by anti-apoptotic proteins like Bcl-xL (purple). Upon a death-promoting stimulus, BAX is activated in both direct and indirect manners. Among the plethora of BAX-activating stimuli, BAX can be directly activated by Ca^2+^ which accumulates due to excitotoxicity in neurons in the penumbra. Indirect activation is mediated by BH3-only proteins here exemplified by BAD (blue). Under physiological conditions, BAD is phosphorylated and therefore unable to bind other Bcl2-proteins. When the cell receives a death stimulus, BAD is dephosphorylated and thereby enabled to bind anti-apoptotic proteins rendering BAX unbound and easily activated (1). Once activated, BAX undergoes a conformational change and integrates into the mitochondrial outer membrane (2) where it forms dimers (3) which further form higher order oligomers (4). BAX oligomerization leads to mitochondrial outer membrane permeabilization (MOMP), releasing cytochrome c and other apoptogenic factors from the mitochondrial intermembrane space into the cytosol. Small molecule protein–protein interaction inhibitors of BAX and BAK have been shown to inhibit formation of higher order oligomers and thereby MOMP, e.g., in excitotoxicity [115]. Of note, even though BAK is not depicted here, BAX and BAK are functionally interchangeable in many tissues and efficient pharmacological inhibition of MOMP would need to target both proteins [115]

Mitochondria are tightly involved in active cell death mechanisms in stroke [6, 31] through the intrinsic apoptotic pathway leading to BAX and BAK dependent MOMP (Fig. 3) and thus cytochrome c release [103, 104]. Further, accumulation of ROS and subsequent mitochondrial permeability transition pore (mPTP) opening or activation of Hypoxia Inducible Factor (HIF)-1α pathways trigger apoptosis. Thereby, mPTP opening leads to elevated calcium release and even higher levels of ROS [105–110]. Especially high amounts of ROS drive a series of downstream effects and act as central regulator. Mitochondria are also involved in other non-apoptotic types of cell death powered by excessive ROS formation like necrosis [111], necroptosis [112], and ferroptosis [113] during reperfusion and pyroptosis in post-ischemic inflammation [108, 114].

#### Regulation of Apoptosis

While necrosis is the predominant cell death mechanism in the ischemic core, cells in the penumbra mainly undergo cell death via apoptosis, and to some extent other types of regulated cell death, which have been reviewed elsewhere [116]. The concept of programmed cell death by apoptosis was first introduced by Kerr et al. in 1972, who described specific morphological changes in dying cells and suggested this mechanism played an opposite role to mitosis [117]. Today, two principal pathways of apoptosis are known: the extrinsic and intrinsic pathway. The extrinsic pathway is mediated by death receptors on the cell membrane that are activated by ligand binding and subsequent caspase activation [105]. The intrinsic or mitochondrial apoptotic pathway relies on the activation of endogenous proteins upon death-promoting signals. The central regulators of the intrinsic apoptotic pathway are members of the Bcl-2 protein family (Fig. 3). Bcl-2 family proteins share one to four Bcl-2 homology (BH) domains [103] and can be divided into anti-apoptotic proteins, pro-apoptotic proteins, and BH3-only proteins. Via the BH-domains, Bcl-2 family members can bind to each other to either promote or prevent cell death [103, 105]. BH3-only proteins detect death stimuli and trigger pro-apoptotic reactions in the Bcl-2 family. Anti-apoptotic proteins such as Bcl-xL compete with the pro-apoptotic proteins to inhibit cell death through direct molecular interactions [118, 119]. The two multi-domain pro-apoptotic proteins BAX and BAK control the point of no return of the intrinsic apoptotic pathway. Whereas BAK is constitutively bound to the mitochondrial outer membrane (MOM), BAX resides in the cytosol in its inactive form and only upon death promoting stimuli it is activated and recruited to the MOM where both proteins lead to formation of homo-oligomers ultimately leading to MOMP in an ordered series of events [120], and the release of apoptogenic molecules like cytochrome c from mitochondria [103]. Critically, targeting BAX and BAK oligomerization in the MOM using small molecule protein–protein interaction inhibitors to prevent MOMP may be an effective strategy to target to prevent cell death in the penumbra [115].

#### Apoptosis in the Penumbra

In the ischemic core, energy failure leads to rapid necrosis within minutes [6]. In contrast, in the penumbra, cell death can continue by apoptosis over a longer period of time [6]. Several mechanisms have been proposed to contribute to this process which are discussed in more detail in the following.

##### Excitotoxicity

When glucose and oxygen are insufficient, energy metabolism in neurons and glia cells is disturbed, which results in decreased ATP production. Hence, neurons and glia cells fail to maintain their ion homeostasis via ATP-consuming proton pumps and their membrane potential is lost [6, 121]. This leads to depolarization of neurons and activation of voltage dependent calcium channels and excitatory neurotransmitters, like glutamate, are released [122]. Furthermore, over-activation of metabotropic glutamate receptors and N-methyl-D-aspartate (NMDA) receptors contributes to accumulation of calcium and further excitotoxicity [19, 121]. Increased glutamate concentration over-stimulates depolarization in neurons and eventually leads to cell death. While excitotoxicity of high magnitude induces rapid necrosis, less strong exposure leads to delayed excitotoxic apoptosis [123], which is mediated by the persistent activation of glutamate receptors due to failures in the energy-dependent re-uptake of the neurotransmitter. This results in increased influxes of Na^+^ and calcium into the cells and to the direct activation of BAX [124] and other apoptosis-related proteins [125, 126]. This insult-evoked cell death can continue for days after the initial insult through apoptotic pathways [116]. However, recently, the role of excitotoxicity in mediating acute brain damage in stroke has been questioned and it has been argued that spreading depolarizations (SD) are the events leading to (neuronal) cell death [127]. However, irrespective of the initiating event (excitotoxicity or SDs), the molecular events that are triggered, including direct activation of BAX and the potential therapeutic implications [115], are likely the same.

##### Spreading depolarization

The term spreading depolarization describes the failure of cellular proton pumps and the breakdown of ion homeostasis and membrane disruption, which disables neurons from generating action potentials [128]. In-turn this leads to cellular depolarization propagating through the grey matter and the contribution to electrochemical membrane failure in the ischemic core [129]. Here, the state of constant depolarization leads to neuronal cell death, likely through calcium-dependent activation of BAX [115, 127], among others. Constant depolarization for more than 15 minutes leads to neuronal cell death in the ischemic core, whereas SD in the penumbra occurs with a delay [127]. Though compromised in function and metabolism, tissue in the penumbra is not yet irreversibly damaged and is therefore able to further spread the depolarization. This phenomenon is termed peri-infarct depolarization [130–132]. Limited access to oxygen due to ischemia increases oxygen demands in the compromised penumbra, which triggers further waves of peri-infarct depolarizations [133].

##### Free Radical Production

In hypoxia, the shift from OXPHOS to glycolysis leads to accumulation of ROS through mechanisms reviewed elsewhere [106], and dysregulation of ionic gradients resulting in membrane depolarization. This further activates voltage-dependent calcium channels allowing release of excitatory amino acids, like glutamate, into the synaptic cleft while energy-dependent reuptake mechanisms are impaired ultimately leading to excitotxicity. The lack of ATP and disturbed electron transport leads to release of free radicals and ROS accumulation [19, 114, 121]. This has a plethora of further downstream effects affecting cell survival by triggering the intrinsic apoptotic pathway leading to MOMP and apoptosis (described above) as well as glucose, lipid, and protein metabolism [106]. Moreover, high levels of ROS can also trigger mitochondrial damage leading to increased mitochondrial fission and mitophagy as well as DNA damage or activation of immune cells, ultimately leading to other types of cell death [89].

##### Reperfusion Injury

In addition to the acute ischemic insult, reperfusion and resulting re-oxygenation cause major injury and lead to the induction of cell death through above mentioned mechanisms. ROS production after reperfusion is highly elevated due to the reverse of the electron transport chain at complex I [114]. In addition, shifting to anaerobic pathways during ischemia leads to succinate accumulation which is rapidly re-oxidized upon reperfusion, leading to even further increase in ROS [111, 114, 134, 135]. In neurons it has been shown that these elevated succinate levels lead to increased mitochondrial fission and dissociation of hexokinase II from mitochondria, subsequently promoting cell death [136].

 The effect of free radicals and reperfusion injury is an example for the tightly regulated balance of homeostasis. While under physiological conditions, ROS protect against oxidative stress, help maintaining the redox equilibrium, act as signaling molecules, and regulate transduction by redox state as well as neuronal activity [24, 137, 138], abnormally high amounts as found in the penumbra, lead to persistent changes in signal transduction and gene expression, initiating a plethora of potentially harmful downstream effects like apoptosis [138, 139]. At what level ROS turn from being necessary and beneficial to generating negative effects like induction of apoptosis is highly relevant but needs further research to understand cell death regulation in the penumbra. Succinate is also present under physiological conditions and is crucial for metabolism and metabolic signaling. Furthermore, it can support immune reactions by stabilizing HIF-1α and promote cytokine secretion [140, 141] described below. However, during stroke, succinate accumulates and significantly contributes to reperfusion injury as described above [135].

Ultimately, the penumbra can be seen as tissue that is prone to apoptosis without having crossed the “point of no return”. For example, BAX/BAK have progressed to the dimer stage but the equilibrium has not yet shifted to formation of oligomers (Fig. 3). Thus, inhibition of BAX/BAK oligomerization or reversal of the equilibrium reactions could promote cell / tissue survival.

## Autophagy

### A Cellular Mechanism to Cope with Adverse Conditions

Autophagy is a cell intrinsic mechanism serving to recycle misfolded proteins, to remove damaged organelles, thereby providing the cell with essential nutrients during nutrient starvation and to eliminate pathogens [142]. Three types of autophagy exist: chaperone-mediated autophagy (CMA), microautophagy, and macroautophagy. All types have in common that their cargo is routed to lysosomes, its place of degradation.

**Fig. 4:**
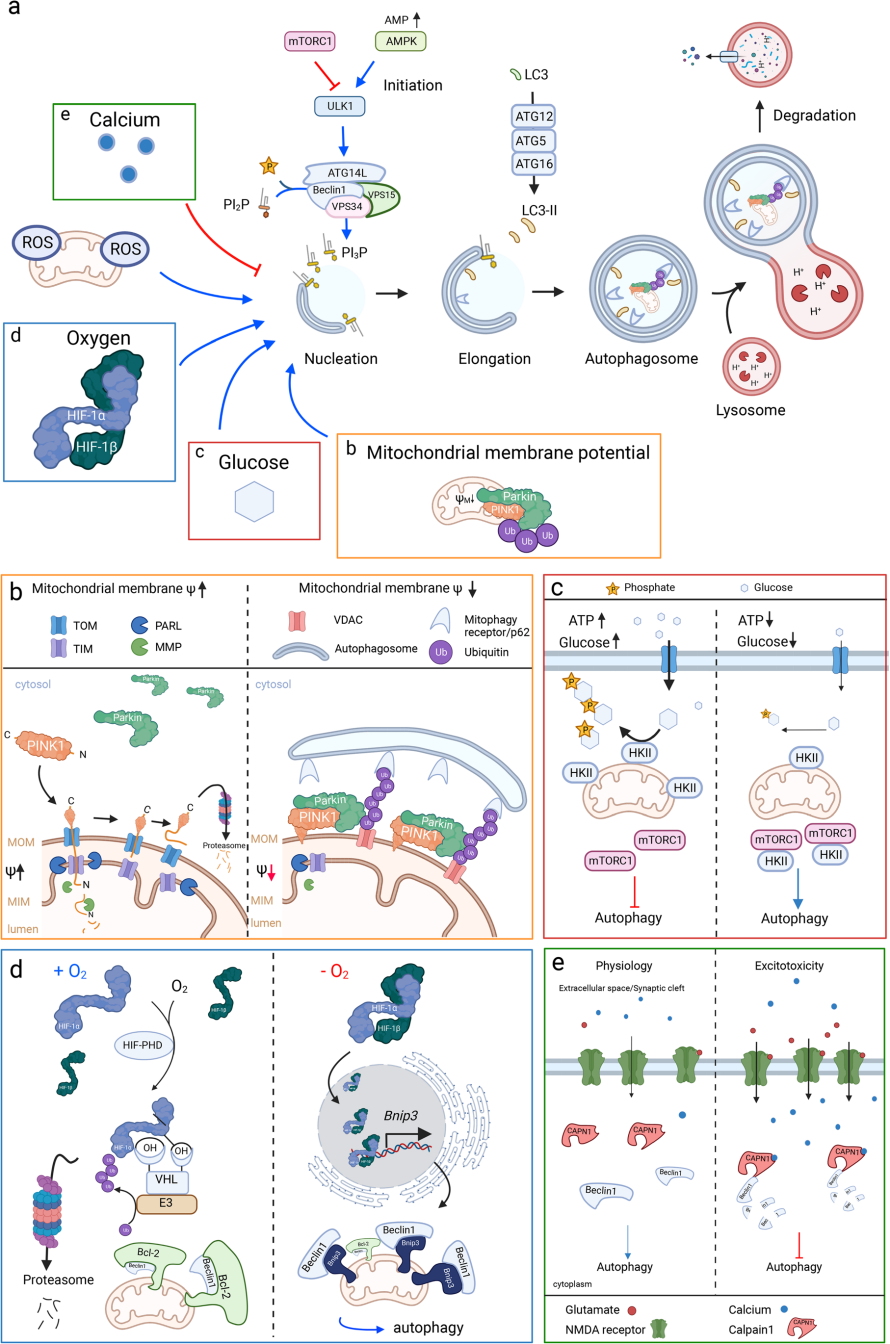
Regulation of autophagy in the penumbra. **a** Autophagosome biogenesis starts with the initiation at the ULK1 complex mediated by an increase in AMP. ULK1 phosphorylates downstream target Beclin1/VPS15/VPS34/ATG14L which is responsible for an increase in phosphatydilinositol-3-phosphate (PI3P). PI3P is crucial for attraction of additional proteins at the phagophore assembly site. Next, the membrane is elongated and adapter proteins like LC3-II are incorporated. Convergence of LC3 to the mature form LC3-II is achieved by ATG12-ATG5-ATG16. Engulfed cargo is enclosed and upon fusion with a lysosome degraded. Oxygen, ROS, glucose, calcium, and the mitochondrial membrane potential (ψM) induce autophagy during ischemia [148]. **b** Mitochondrial membrane potential is required for the degradation of PINK1. Under physiological conditions, the mitochondrial membrane potential leads to the mitochondrial import of PINK1 through translocase of the outer membrane (TOM) and translocase of the inner membrane (TIM). PINK1 is cleaved by presenilin associated rhomboid-like protein (PARL) and mitochondrial matrix metalloproteinases (MMP). During ischemia, the mitochondrial membrane potential collapses and PINK1 is stabilized at the mitochondrial outer membrane (MOM), Parkin is recruited and ubiquitinylates VDAC, among others. The adaptor protein p62 recognizes ubiquitinylated VDAC and the autophagosome starts to form and engulf the damaged mitochondrion [149, 150]. **c** Autophagy induction is thought to be mediated by hexokinase II under glucose-deprived conditions. When glucose is absent, HKII detaches from mitochondria and binds to mTORC1, thereby releasing its suppressive role on autophagy. **d** When oxygen is available, HIF-PHDs hydroxylate HIF-1α at two proline residues under oxygen consumption. Prolylhydroxylated HIF-1α is recognized by the von-Hippel-Lindau (VHL) protein and ubiquitinylated by E3 ubiquitin ligases and degraded by the proteasome. Beclin1 is bound by Bcl-2 which antagonizes the autophagy inductive function of Beclin1. When oxygen availability is limited, HIF-1α forms a heterodimer with HIF-1β and translocates to the nucleus. HIF-1 binds to the hypoxia response element of the BNIP3-gene, among others, and leads to transcriptional upregulation of Bnip3. After translocating to the MOM, Bnip3 initiates mitophagy. **e** Increased concentrations of glutamate overactivate NMDA-channels and lead to intracellular calcium accumulation. After stroke, calcium activates Calpain1 which cleaves Beclin1 and thereby suppresses autophagy [151]

Chaperone-mediated autophagy promotes degradation of proteins with a KFERQ-motif sequence by heat-shock cognate 71-kDa protein 70 (HSC70) trafficking across the lysosomal membrane, its place of digestion [143, 144]. Microautophagy refers to membrane engulfment of proteins, lipids, and organelles that are routed to members of the endosomal pathway like late endosomes and multivesicular bodies, before being degraded in lysosomes [145]. Macroautophagy, hereafter referred to as autophagy, is characterized by the formation of an autophagosome with two surrounding membranes that engulf the cargo destined for degradation. Once formed, autophagosomes fuse with lysosomes to become autolysosomes. In addition to bulk autophagy, adapter proteins mediate engulfment of damaged organelles and substrates leading to cargo-specific turnover [146, 147] (Fig. 4a).

Mitophagy refers to the degradation of mitochondria, which is mediated by autophosphorylation of PINK1 that is stabilized on the MOM of depolarized mitochondria and phosphorylates the ubiquitin ligase Parkin which facilitates ubiquitination of the mitochondrion and thereby marking it for translocation to the autophagosome [152, 153]. Similarly, other damaged organelles are degraded by endoplasmic reticulum (ER)-phagy [154, 155] or ribophagy [156]. Lipophagy is a type of selective autophagy where denatured lipids are stored in lipid droplets and serve as substrate for β-oxidation, contributing to the energy homeostasis [157]. Aggregated proteins, as can be observed in neurodegenerative diseases, are cleared by aggrephagy via ubiquitination and autophagosomal translocation [158].

Maintaining homeostasis in neurons is essential for neurotransmission and control of presynaptic vesicle release involves autophagy [159]. For example, knock-out of autophagy-related 5 (ATG5), which is essential for autophagosome formation, leads to dysregulated excitatory neurotransmission. In the ATG5 knockout, extensions of the endoplasmic reticulum protrude into axons, and excitatory neurotransmission facilitated by calcium release from the extended ER was observed. This exemplifies the role of autophagy in axonal calcium storage and neurotransmission [160]. Furthermore, at least in drosophila, autophagy-regulated signaling has also been implicated in memory formation [161].

### Autophagy in Cerebral Ischemia

Autophagy is a hallmark of all cells affected by ischemia and plays an essential role in the shift of metabolism, the induction of apoptosis and cellular homeostasis. Understanding the impact of autophagy timing and degraded cargo in a cell type-specific manner towards the balance of cellular survival and death might render new targets for intervention.

In cerebral ischemia, cells suffer from severe nutrient deprivation and organelle damage. In consequence, autophagy is upregulated during ischemia [162]. Immediately after reduction of glucose and oxygen availability, the autophagy machinery is activated, indicated by an immediate increase in LC3-II:LC3-I ratio that declines in the following hours but stays elevated compared to normoxic conditions in rat hippocampal neurons subjected to 1 hour of OGD/R [163]. In the same study, autophagic flux, measured by abundance of the autophagy adaptor protein p62/SQSTM1, was found strongly increased after OGD, but decreased even below normoxic rates after 12 and 24 hours of OGD/R [163]. Prolonged upregulation of autophagic markers has been found in vivo up to at least 6 days after one hour of transient middle cerebral artery occlusion (tMCAO) in a mouse model of stroke [162]. Together, these findings highlight the dynamic nature of autophagy after cerebral ischemia.

Induction of autophagy was also found in hippocampal CA1 cells in a rat model of global ischemia by a decrease of intracellular concentration of mammalian target of rapamycin (mTOR) [164]. Furthermore, Hamartin induces autophagy and is selectively upregulated in hippocampal CA3 neurons in contrast to CA1 neurons upon stroke via an mTOR complex (mTORC)1-dependent mechanism. Hamartin was shown to be elevated after ischemic preconditioning in CA1, thereby suggesting a mechanism for preconditioning protection [165].

Autophagy induction via delta opioid receptor agonist was reported to confer neuroprotection in CA1 neurons through activation of the autophagic 5' adenosine monophosphate-activated protein kinase (AMPK) / mTOR / Unc-51-like kinase 1 (ULK1) pathway [166]. Furthermore, ischemic preconditioning in a rodent model with short periods of ischemia before permanent vessel occlusion showed reduced neuronal death while autophagy levels were increased [167]. Increased mitophagy in neurons and to a lesser extent in astrocytes and endothelial cells via the PINK1/Parkin axis peaking 24 hours after reperfusion was reported after MCAO in rats [149]. Loss of mitochondrial membrane potential observed in stroke leads to stabilization of PINK1 on the MOM and mitophagy [149] (Fig. 4b).

In stroke, glucose availability of affected cells is dramatically decreased leading to a change in metabolism that is integrated with autophagy by HKII (Fig. 4c). When glucose is limited, HKII binds to the master regulator of autophagy mammalian target of rapamycin complex 1 (mTORC1) [168]. mTORC1 suppresses the kinase activity of ULK1, which in turn is necessary for autophagosome formation [169]. By binding to mTORC1, HKII releases the break on autophagy under glucose-deprived conditions [168]. Furthermore, HKII appears to be degraded by CMA when it does not bind glucose [170].

Reduction of oxygen availability is integrated with autophagy by HIF-1 signaling (Fig. 4d) via Beclin1 and BNIP3 (B cell leukemia/lymphoma-2/adenovirus E1B 19 kDa protein-interacting protein 3) [171, 172]. BNIP3 was found to be involved in clearance of damaged mitochondria by mitophagy [173]. Hypoxic preconditioning induced HIF-1α-dependent upregulation of Beclin1 and BNIP3 in SH-SY5Y neuroblastoma cells, enhancing autophagy and attenuating OGD/R-induced apoptosis [174]. On the other hand, following stroke, autophagy may be downregulated with Calpain1 dependent cleavage of Beclin1, mediated by intracellular accumulation of calcium, leading to suppression of autophagy in a permanent MCAO model of stroke [151] (Fig. 4e).

Furthermore, autophagy induction triggered by ROS was shown to be mediated by ataxia-telangiectasia mutated kinase (ATM) acting as ROS sensor subsequently phosphorylating cell cycle checkpoint kinase 2 (CHK2), which in turn activates Beclin1 by phosphorylation. Induction of autophagy via ATM / CHK2 / Beclin1 axis contributes to cell survival observed in a tMCAO mouse model of stroke suggesting an important role for autophagy in maintaining ROS homeostasis [175].

During serum starvation, increased levels of autophagy protect the BBB by scavenging ROS and inhibiting detrimental reorganization of the tight junction protein Claudin-5 [176, 177]. Furthermore, the autophagy enhancing long noncoding RNA Malat1 was found to increase survival of BMVEC during OGD/R [178]. Furthermore, remodeling of lipids under oxidative stress is induced by ischemia–reperfusion and reshaping of lipids together with biogenesis of lipid droplets was observed after OGD in rat brain endothelial cells [179]. Lipid droplets were found to co-localize with the autophagosomal marker LC3, suggesting selective activation of lipophagy upon ischemia [179].

Microglia polarization towards a pro- or anti-inflammatory phenotype is accompanied by decreased or increased levels of autophagy. Microglia overexpressing peroxisome proliferator-activated receptor gamma coactivator 1-alpha (PGC-1α), which acts upon ULK1 to enhance autophagy, showed decreased pro-inflammatory response after tMCAO in a rodent model of stroke with improved neurological outcomes [180]. Sestrin2 was reported to inhibit mTOR leading to increased autophagic flux and anti-inflammatory polarization of microglia with improved neuronal survival after tMCAO [181]. Further anti-inflammatory influence of microglial autophagy is demonstrated by the observation of selective degradation of IKKα, resulting in downregulated NF-κB signaling, anti-inflammatory microglia phenotype polarization, and improved neurological outcome after ischemia [182].

However, some authors reported overactive autophagy to promote cell death in ischemia as a distinct cell death pathway [183]. Autophagic cell death is not trivial to investigate, as chemicals used to prevent autophagy have proven to exert additional autophagy-independent regulatory mechanisms on cell death pathways [184–186]. Future studies will need to carefully disentangle the various, sometimes counteracting cellular pathways that occur simultaneously.

Thus, to exploit autophagy in order to rescue penumbral tissue at risk after stroke, it is necessary to investigate the spatiotemporal regulation of autophagy in a cell type-specific manner. Additionally, severity of oxygen and glucose deprivation must be considered as well as the spatial distribution of individual cells within the penumbra in in vitro and in vivo models to achieve translational success.

## Neuroinflammation After Stroke

### The Neuroinflammatory Cascade

**Fig. 5:**
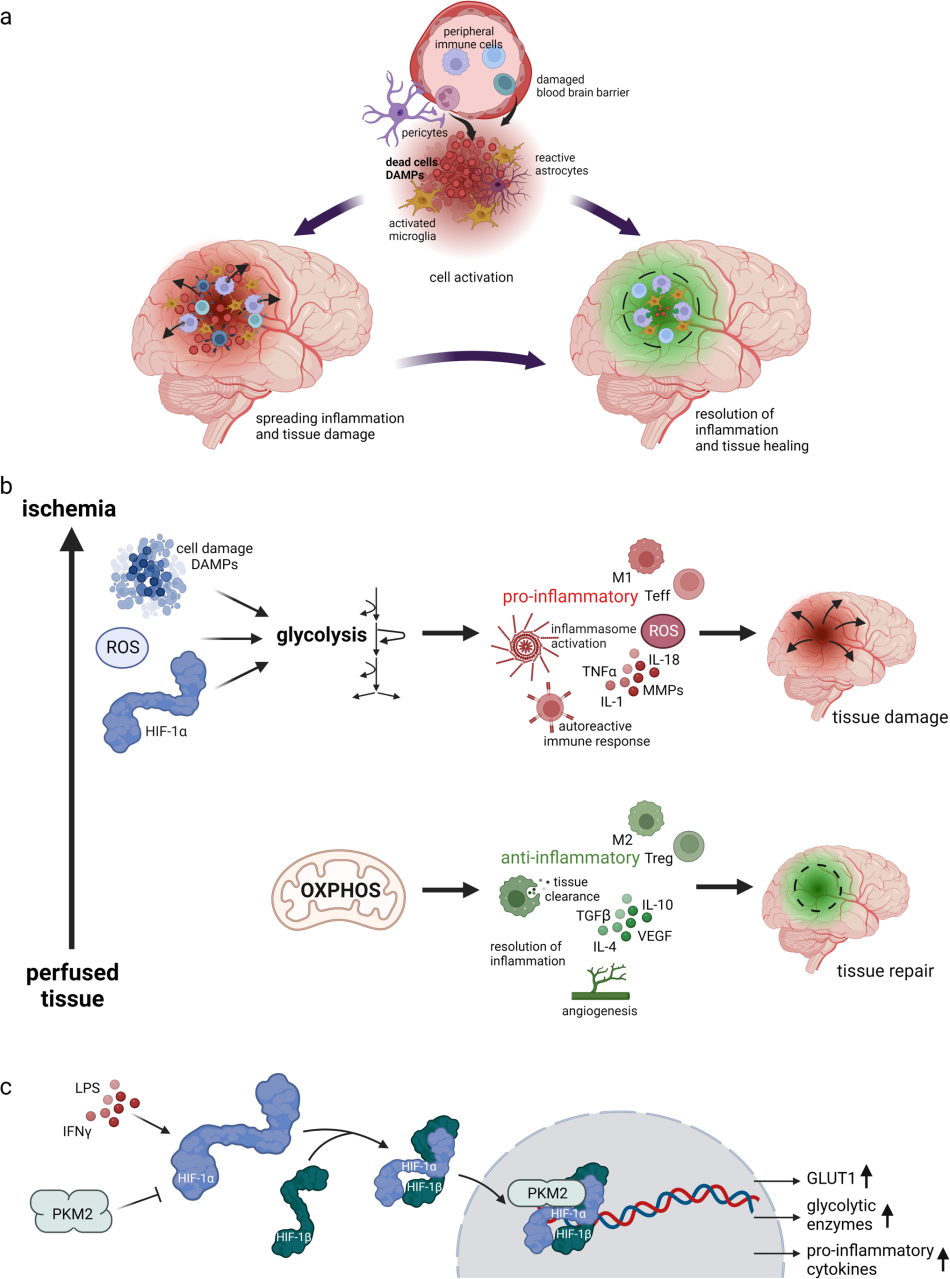
**a** Immune cells contribute to the neurodegenerative outcome after stroke. Ischemic brain injury induces rapid cell death in the stroke core, which activates microglia and astrocytes. Furthermore, the blood brain barrier (BBB) is disrupted which promotes infiltration of peripheral immune cells into the brain, mediated by release of chemoattractants and DAMPs from the injured area. Immune cells in the brain polarize to a pro- or anti-inflammatory state and thereby can have a dual effect on the neurodegenerative outcome after stroke by promoting spreading cell death or tissue healing. **b** Immune cell glucose metabolism regulates immune cell function. In stroke, the ischemic environment promotes a metabolic shift towards glycolysis by upregulation or release of pathological effectors. High levels of glycolysis are strongly connected to M1 macrophage polarization and effector T (T_eff_) cell function, which have a pro-inflammatory phenotype, except for Th2 cells. Pro-inflammatory immune cells exacerbate inflammation, BBB breakdown, and autoreactive immune response, which aggravates acute neurodegeneration. On the contrary, under non-ischemic conditions, immune cells preferentially perform OXPHOS, which is a main energy source for anti-inflammatory immune cells like M2 macrophages or Tregs. These cells promote tissue clearance, angiogenesis and resolution of inflammation, thereby contributing to tissue repair. **c** HIF-1α signaling may be regulated by cytokines and PKM2 in macrophages. LPS and IFNγ stimulation promote M1 polarization and HIF-1α signaling. Moreover, dimeric PKM2 has been suggested to act as a co-activator for HIF-1α signaling, whereas tetrameric PKM2 is thought to inihibit it. However, the exact mechanism of PKM2 co-activator activity is not well established. HIF-1α is a transcription factor for GLUT1, glycolytic enzymes, and pro-inflammatory cytokines, which highlights the strong connection between oxygen supply, glucose metabolism, and immune cell function

Brain tissue damage in stroke triggers activation of microglia, neuroinflammation, and the breakdown of the BBB [187] (Fig. 5a). Furthermore, recruitment of peripheral immune cells can exacerbate BBB disruption and neuronal injury. Early immunological events include complement activation, neutrophil infiltration, and myeloid cell accumulation in the brain within the first hours and days after the insult [188, 189], followed by lymphocyte infiltration [188]. After stroke, immune cells can adopt neuroprotective or neuro-destructive phenotypes, which can modulate the neurodegenerative damage [190]. The different mechanisms have been extensively reviewed elsewhere [187, 188, 191].

### Spreading Inflammatory Neurodegeneration

Several immunological events orchestrated by different cell types contribute to or dampen spreading cell death after stroke. In the penumbra, neuroinflammatory events and microglia and astrocytes shape tissue damage in the days and weeks after stroke [191, 192]. Astrocytes form glial scars after CNS injury restricting the spread of neuroinflammation [193]. Transgenic ablation of reactive astrocytes prolonged leucocyte infiltration and BBB disruption, and enhanced neuronal degeneration following forebrain stab injury in mice [194]. Therefore, astrocyte compartmentalization might act as an important mechanism to slow down spreading inflammation.

Ischemic neurons release damage associated patterns (DAMPs) initiating sterile inflammation and activating phagoptosis mediated by microglia [195, 196]. Mice lacking the phagocytic receptor Mer receptor tyrosine kinase (MerTK) showed improved neuronal survival and stroke outcome [195, 197]. On the contrary, microglial deficiency of the phagocytic triggering receptor expressed on myeloid cells-2 (TREM2) was found to worsen neurological recovery and increase infarct size in mice following distal MCAO [198].

Microglia and macrophages can contribute to tissue clearance, angiogenesis, and resolution of the inflammatory state [188, 199–202]. Similar to myeloid cells, infiltrating T lymphocytes can become pro- or anti-inflammatory, thereby accelerating neurotoxicity or contributing to neuroprotection [187]. Furthermore, in the late phase after stroke, B cell clusters can form in the brain that can produce autoreactive CNS specific antibodies. Thus, activation of the adaptive immune system can promote either an autoreactive tissue damage or contribute to resolution of inflammation [187, 188, 203, 204]. Neuroinflammation might also contribute to spreading cell death in the metabolically compromised penumbra. This is supported by data from a rat model of permanent focal ischemia where neuroinflammation associated with a high metabolic rate was observed in the peri-infarct area compared to the stroke core [205].

Stroke can also lead to systemic immunodepression, which promotes high susceptibility to following infections such as pneumonia [206]. Both immune exhaustion and dysregulation of the sympathetic nervous system have been implicated in the cause of this immunodepression [207, 208]. Therapeutic regulation of immune cell behavior might thus not only be advantageous to reduce brain damage after stroke but could also be a strategy to prevent secondary diseases [209]*.*

### Metabolic Regulation of Immune Cells

Oxygen and nutrient supply influence immune cell metabolism, which is strongly coupled to their function and activation state [210, 211]. In particular, glucose metabolism plays a central role in immune cell polarization [212] (Fig. 5b). In stroke-induced neuroinflammation, most effector T cells like Th1, Th17, and cytotoxic T cells promote neurotoxicity, except for Th2 cells, which are associated with neuroprotection [203]. In general, pro-inflammatory microglia, macrophages, and effector T cell subsets show high aerobic glycolytic activity [213], while resting T cells, regulatory T cells (Tregs), and anti-inflammatory microglia and macrophages predominantly perform OXPHOS [213]. Strikingly, glucose metabolizing enzymes can directly regulate immune cell function. For example, glyceraldehyde-3-phosphate-dehydrogenase (GAPDH) can repress interferon-gamma (IFNγ) translation in T cells. However, engaging GAPDH in the glycolysis pathway increases IFNγ levels [214].

mTOR is a key regulator of immune cell function, which couples environmental changes and cytokine signaling with metabolism in the context of immune cell function [215]. Naïve T cells and anti-inflammatory Tregs mainly perform fatty acid oxidation and OXPHOS, and in these cells mTOR is downregulated [216]. On the contrary, mTOR activation is associated with upregulation of glycolysis in immune cells [215, 217]. Interestingly, in cytotoxic T cells, glucose deprivation activates AMPK, which is a negative regulator for mTORC1 signaling [218]. Furthermore, 2-deoxyglucose (2-DG) stimulation or treatment with the mTOR inhibitor rapamycin promoted Treg differentiation and inhibited Th17 differentiation from naïve T cells [219]. Moreover, mTOR upregulates the transcription factors Myc and HIF-1α in T cells [220]. Interestingly, many glucose metabolizing enzymes and pro-inflammatory cytokines like IL-1β are HIF-1α target genes [221] and HIF-1α is involved in activation of pro-inflammatory Th17 cells, macrophages, and microglia [219, 221–223].

Similar to T cells, macrophages strongly couple glucose metabolism to immune cell function. Dimeric PKM2 appears to promote expression of glycolytic enzymes and cytokines together with HIF-1α, although the exact mechanism is not clear [38, 224]. PKM2 has mainly been implicated in proliferation [38], but induced tetramerization of PKM2 has been shown to prevent HIF-1α from binding to the pro-inflammatory IL-1β promotor and promote anti-inflammatory gene expression in lipopolysaccharide (LPS) treated macrophages [225]. Furthermore, in pro-inflammatory M1 polarized macrophages, the TCA cycle is interrupted, which leads to succinate accumulation. Succinate inhibits prolyl hydroxylases (PHDs) and thereby promotes HIF-1α stabilization and IL-1β expression [141, 221]. On the contrary, the TCA cycle metabolite itaconate has been shown to control the inflammatory phenotype of macrophages with mainly regulating anti-inflammatory function [226] by inhibiting succinate dehydrogenase in neurons, among others [227]. Furthermore, itaconate administration has been shown to decrease reperfusion injury in mouse models of myocardial infarction [227] and stroke [228], however, without demonstrating itaconate directly impacting immunological mechanisms.

Moreover, ROS and glucose metabolizing enzymes like HK and PKM2 can promote pro-inflammatory NLRP3 inflammasome activation [229–231]. PKM2 upregulation has also been found in neutrophils after stroke in humans and mice, and improved outcome has been observed after deletion of PKM2 in myeloid cells in several mouse models of stroke [232]. However, since deletion of PKM2 affected all myeloid cells, this study [232] could not rule out that the observed improvement in outcome was due to inflammatory regulation of macrophages. In a mouse model of Alzheimer's disease (AD), pharmacological or genetic inhibition of PKM2 decreased microglial activation and improved spatial learning and memory [233].

Systemic inflammatory stimuli and infection can modulate the inflammatory response of innate and adaptive immune cells [191, 234, 235]. In microglia, this can induce innate immune memory and epigenetic immune tolerance which can last up to 6 months. Repeated pro-inflammatory LPS treatment induced immune tolerance, lead to downregulation of HIF-1α and glycolysis, and reduced neurodegeneration in a mouse model of AD [235]. In contrast, onetime LPS stimulation induced immune training, which increased plaque areas, promoted HIF-1α activation in plaque-associated areas, and upregulated glycolytic activity in wild type and Alzheimer’s disease mice [235]. Moreover, LPS treatment prior to induction of stroke reduced neuronal damage and microglia activation in mice [235]. In addition, increased OXPHOS in microglia was associated with higher phagocytic function and improved stroke recovery in mice [236]. Together, this highlights the impact of glucose metabolism on the regulation of neuroinflammation in neurodegenerative diseases.

Furthermore, preliminary evidence reported in a recent preprint indicates that ischemia triggers lipid accumulation in microglia of young mice, promoting premature aging of microglia [237]. Lipid droplet accumulation has been reported to be a feature of aged microglia [238] and it has been suggested that after stroke, an increase of lipid droplets in microglia may contribute to poor stroke outcome in old mice [237]. Given that this effect might be attributable to phagocytosis of cell debris due to brain injury leading to intracellular accumulation of lipids in microglial cells, this further links the inflammatory response in stroke to mitochondrial metabolism as well as lipophagy (see above).

Despite the increasing knowledge about metabolic control of peripheral innate and adaptive immune cells, very little is known about metabolic regulation of immune cells in the brain in stroke. Yet, it is mechanistically quite conceivable that the metabolically compromised environments of the stroke core and the penumbra impact immune cell metabolism and thereby contribute to driving neuroinflammation after stroke.

To reduce neuroinflammatory damage and take advantage of the healing potential of the immune system, it is necessary to investigate the regulation and the effect of immune cells in the penumbra. For this, spatially resolved studies are needed, that consider the high plasticity of immune cells, multi-cellular crosstalk and metabolic regulation in a time-dependent manner. Thus, a better understanding of the mechanisms of metabolic immune cell regulation in the brain will help defining targets for the development of novel therapeutic approaches.

## Concluding Remarks

Our modern understanding of the penumbra is shaped by knowledge of a wealth of molecular and cellular events discovered since the inception of the original concept. Even though early work had measured metabolism in the penumbra with positron emission tomography [239, 240], clinical application of the concept of the penumbra has largely focused on blood flow and the resulting functional tissue failure. Metabolic magnetic resonance imaging [241–243] now has the prospect of establishing metabolic studies in the clinical care of stroke patients and may be a tool for monitoring the health of the penumbra in clinical practice. However, ultimately, we posit that successful translation will only arise if a “penumbral state” can be defined for each of the different mechanisms that work together in concert to define the penumbra at large. We acknowledge that methods for multiparametric analysis in preclinical models as well as for monitoring biomarkers in clinical practice will be the prerequisite for successful neuroprotection translation in the future.

## Data Availability

Not applicable.
